# A quantitative method for the determination of rock fragmentation based on crack density and crack saturation

**DOI:** 10.1038/s41598-023-38911-2

**Published:** 2023-07-20

**Authors:** Yao Xiao, Huafeng Deng, Jianlin Li, Meiling Zhou, Eleyas Assefa, Xingzhou Chen

**Affiliations:** 1 Key Laboratory of Geological Hazards on Three Gorges Reservoir Area (China Three Gorges University), Ministry of Education, Yichang, 443000 Hubei China; 2 College of Architecture and Civil Engineering, Addis Ababa Science and Technology University, Addis Ababa, Ethiopia; 3 College of Architecture and Civil Engineering, Xi’an University of Science and Technology, Xi’an, 710054 Shanxi China

**Keywords:** Civil engineering, Mechanical engineering

## Abstract

Rock mechanics tests are essential for advancing theoretical and practical knowledge in the field. The rock failure mechanism can be studied by analyzing the failure characteristics of rock samples through mechanical tests. However, despite their usefulness, quantitative rock classification systems still possess certain limitations that need to be addressed. The main objective of this paper was to develop a comprehensive quantitative rock classification system based on rock failure characteristics. The rock classification indices, including crack density and crack saturation, were systematically introduced based on rigorous statistical analyses conducted on a diverse set of 200 rock samples. In particular, the crack saturation index serves as a crucial metric that primarily captures and quantifies the extent of actual crack propagation within the rock samples. Moreover, it is important to note that the two evaluation indices, crack density and crack saturation, work in harmony and complement each other, enhancing the overall understanding of rock fragmentation and failure characteristics. By taking into account both crack density and crack saturation, the proposed method effectively categorizes rock fragmentation into five distinct classes, namely “relatively intact”, “slightly fragmented”, “fragmented”, “very fragmented” and “extremely fragmented.” The validation process confirmed the efficacy of the proposed classification method in accurately capturing the crack-propagation characteristics of rocks. This outcome is highly significant as it significantly advances ones understanding of rock failure mechanisms and provides valuable insights into the overall characteristics of rocks.

## Introduction

In addition to their practical applications in evaluating mechanical parameters, rock mechanics tests also hold considerable relevance in advancing theoretical research within the field^1–3^. Despite the advancements made in theoretical and numerical analyses of rocks, the significance of rock mechanics tests remains paramount in effectively addressing and solving practical problems encountered in the field^4,5^. As research topics continue to advance, the necessity for meticulous analysis and interpretation of high-quality test results becomes imperative. Specifically, the determination of strength and deformation characteristics is crucial for a comprehensive understanding and accurate characterization of a specific rock mass. These parameters play a pivotal role in assessing the behavior and response of the rock mass under various loading conditions. However, it is important to recognize that during the construction and operation of an engineering project, the deformation characteristics of the rock mass have a profound impact on its stability. Properly understanding and considering these deformation characteristics are vital for ensuring the long-term stability and integrity of the rock mass within the project. Therefore, the study and understanding of rock deformation and failure characteristics hold paramount importance in advancing both theoretical knowledge and practical applications within the field of rock mechanics.

In practical applications, the integrity of rock formations is heavily influenced by the presence and behavior of joints and fissures. These geological features play a crucial role in determining the stability and overall mechanical behavior of the rock masses. In order to assess the integrity of rocks, specifically their fragmentation degree, Müller introduced a classification table that incorporates two evaluation indices, namely fissure degree and cutting degree. This classification system provides a framework for evaluating and categorizing the extent of fragmentation within rock formations. Classification methods have been developed based on a wide range of engineering projects, including but not limited to underground structures, slope stability problems, water conservancy projects, and hydropower projects. These methods are tailored to address the specific challenges and requirements of each project type, facilitating effective decision-making and risk assessment in engineering design and construction processes. Over time, the classification approach has undergone several developmental processes, transitioning from an initial single-indicator qualitative approach to a widely adopted comprehensive multi-indicator quantitative approach. These modifications reflect the evolving needs of the field, emphasizing the importance of incorporating multiple indicators to achieve a more accurate and comprehensive assessment of rock characteristics and failure behavior. Several established rock classification systems exist, such as RQD (Rock Quality Designation)^6^, RMR (Rock Mass Rating)^7^, RMI (Rock Mass Index)^8^, Q-value^9^, and GSI (Geological Strength Index)^10^. In particular, Deere^6^ introduced the RQD index, which assesses rock quality based on core recovery and quality, contributing to the overall understanding and categorization of rock masses. In 1974, Bieniawski introduced a standardized technique known as rock mass rating (RMR). This technique has been widely employed to systematically evaluate the quality of rock mass, providing valuable insights into its overall stability and suitability for engineering applications. The RMR classification system encompasses six key parameters that include uniaxial compressive strength, Rock Quality Designation (RQD), discontinuity spacing, discontinuity condition, discontinuity orientation, and groundwater conditions. These parameters collectively contribute to a thorough evaluation of rock mass characteristics, providing valuable insights into its mechanical properties, stability, and potential engineering challenges. According to the RMR system, rock quality is classified into five distinct categories, spanning from “very good” to “very poor”. This classification scheme allows for a comprehensive assessment of the overall quality and suitability of the rock mass for engineering purposes, providing a basis for informed decision-making in construction and design projects. In the same year, Barton developed a rock mass classification system specifically tailored for underground structures. This classification system was formulated based on an in-depth analysis of tunneling case studies in Scandinavian countries. By incorporating the practical experiences and challenges encountered in these projects, Barton’s classification system provides a valuable framework for assessing and addressing the geotechnical aspects of underground construction projects. Considerable research on rock-mass classification systems has been undertaken in China as well. The scientific community has actively contributed to the development and refinement of classification systems, taking into account the unique geological and engineering challenges encountered in various regions of the country (For example, a national standard for rock mass classification (GB/T 50218-2014)^11^). This standard employs either elastic wave velocity or volumetric joint count as metrics to evaluate the quality of a rock mass.

Rock samples demonstrate the presence of cracks and irregular fragmentation in varying sizes and shapes when subjected to uniaxial or triaxial loading and unloading during mechanical tests^12,13^. The failure characteristics exhibited by rock samples are intricately influenced by various factors, including the rock type as well as the specific loading and unloading stress paths applied. Conducting an accurate analysis of failure characteristics is of utmost importance as it allows for a comprehensive understanding of the failure mechanism and mechanical properties of rocks under various stress paths^14–16^. Previous studies have put forth systematic classifications of failure modes, including shear or tension failure, by considering the direction and angle of crack propagation. Nevertheless, when it comes to the fragmentation degree, it has been primarily described in qualitative terms such as “comparative fragmentation” and “fragmentation.” However, there remains a significant gap in terms of quantitative evaluation indices that can precisely quantify and measure the extent of fragmentation^17,18^. The quantitative evaluation and comparison of failure states under different loading and unloading stress paths pose significant challenges across various types of rocks. This limitation hinders the accurate description of failure characteristics of rock samples through mechanical tests.

These indicators hold widespread acceptance and utilization in the field of rock mechanics, serving as a solid foundation for the evaluation of rock mass quality. However, it is important to acknowledge that these indicators do have limitations when it comes to the quantitative analysis of small-scale rock samples. Therefore, it is essential to establish a specialized set of indicators and systems that are specifically designed and optimized for assessing the degree of fragmentation in small-scale rock samples. Following a rigorous statistical analysis of multiple laboratory tests, this study presents a quantitative classification method for evaluating the degree of rock fragmentation.

## Classification indices of rock fragmentation

### Crack density

**Figure 1:**
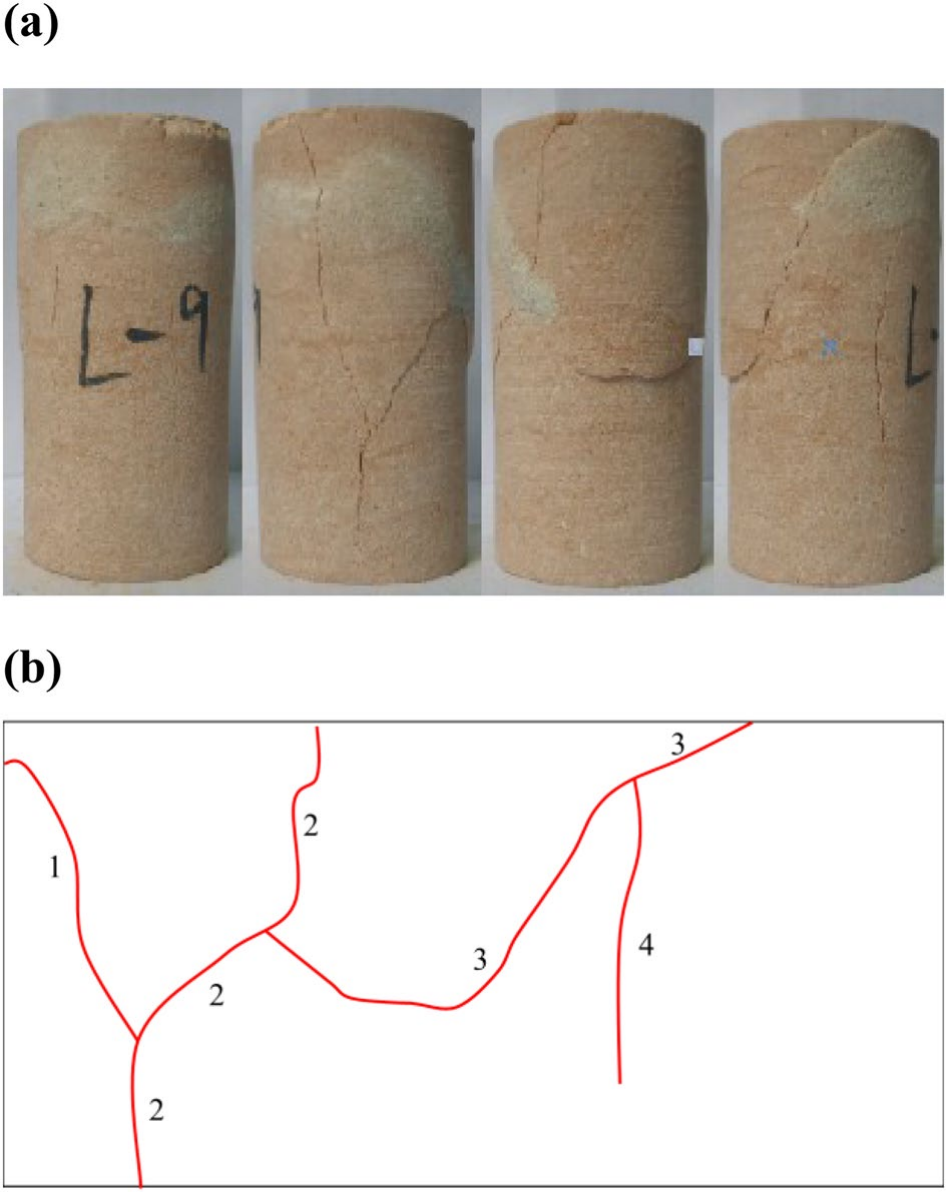
Crack propagation diagrams for typical rock samples: (**a**) Crack morphology of rock samples, (**b**) crack propagation.

Initially, a crack propagation diagram was constructed for a standard cylindrical rock sample with dimensions of 5 cm in diameter and 10 cm in height, as illustrated in Fig. 1. Subsequently, an in-depth analysis was conducted on the crack distribution characteristics, degree of fragmentation, and fracture modes of the rock samples, utilizing existing literature as a reference (Hallbauer et al.^19^; Tulinov et al.^20^). A close relationship between the number of cracks and the degree of fragmentation was observed, as depicted in Fig. 1. The number of cracks and degree of fragmentation were closely related (Fig. 1). Based on the observed relationship, the fragmentation degree can be accurately defined and quantified in terms of the number of cracks present in the rock samples. To quantify the crack density, it was determined by expressing the ratio of the number of cracks to the area along the side of the rock sample. This relationship is mathematically represented by Eq. (1):

1 $$\Gamma_{{\text{J}}} = K \cdot \frac{{Q_{J} }}{S}.$$

In the aforementioned equation: $\Gamma_{{\text{J}}}$ represents the crack density (strip/cm^2^), $Q_{{\text{J}}}$ denotes the number of cracks (strips), S signifies the unfolded area along the side of the rock sample (cm^2^), and K denotes the adjustment coefficient.

**Figure 2:**
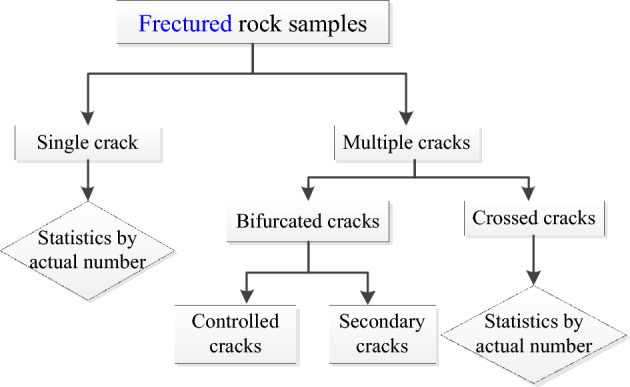
Statistical flow chart for crack number.

**Figure 3:**
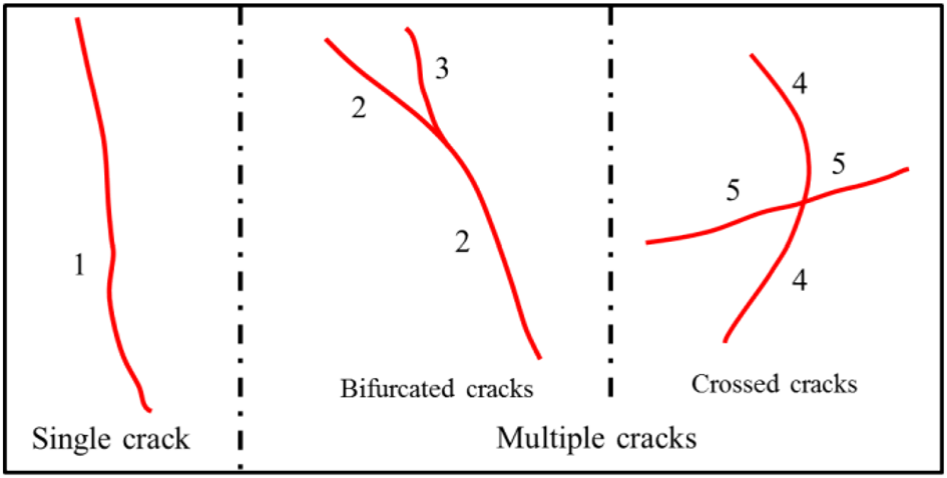
Different types of rock cracks.

To ensure consistency in the analysis, the following steps were implemented: Firstly, the counting of cracks was performed individually, ensuring that each crack was considered separately. Secondly, in cases where crack bifurcation occurred, secondary cracks were counted sequentially after the primary, continuous crack had been accounted for. The cross-cracks were meticulously counted based on their specific direction of propagation. Figure 2 illustrates a flowchart representing the step-by-step process of the statistical analysis conducted in this study. Moreover, Fig. 3 provides a schematic representation of the different types of cracks observed in the rock samples.

**Figure 4:**
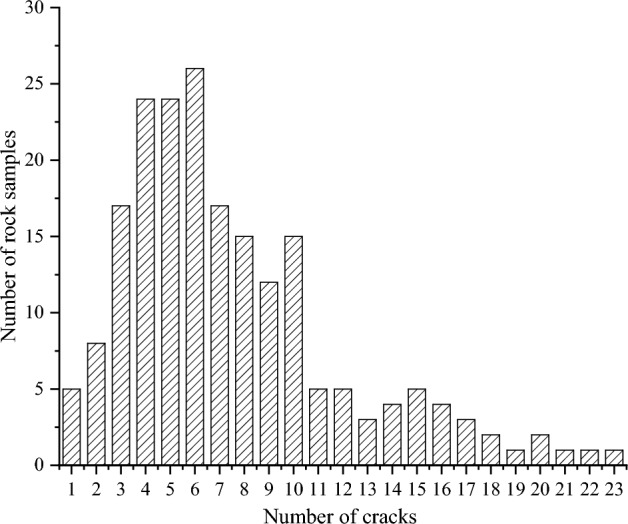
Histogram of fractured rock samples.

In addition to utilizing data obtained from the literature, this study incorporated a comprehensive dataset comprising 200 rock samples of various lithologies, including sandstone, limestone, granite, mudstone, and gneiss. The number of cracks present in the rock samples was counted, as depicted in Fig. 4. Statistically, it was observed that rock samples exhibited significant damage when the number of cracks exceeded a threshold of 20. To facilitate comparison, a crack density of 1.00 was assigned for the case involving 20 cracks. Therefore, the value of K can be determined as $K = \left( {D/2} \right)\pi$ (where D represents the diameter of the rock sample in centimeters). For instance, when considering a diameter of 5 cm, the value of K is determined to be 2.5π. The calculated crack densities for different samples can be found in Table 1, providing a quantitative measure of the concentration of cracks. Table 2 presents the crack densities of the 200 rock samples, calculated using Eq. (1).

**Table 1:** Crack density of rock samples.

Crack number	1	2	3	4	5	6	7	8	9	10	11	12
Crack density	0.05	0.10	0.15	0.20	0.25	0.30	0.35	0.40	0.45	0.50	0.55	0.60
Crack number	13	14	15	16	17	18	19	20	21	22	23	…
Crack density	0.65	0.70	0.75	0.80	0.85	0.90	0.95	1.00	1.05	1.10	1.15	…

**Table 2:** Statistical data of crack density.

Crack density	0–0.10	0.11–0.20	0.21–0.30	0.31–0.40	0.41–0.50	0.51–0.60	0.61–0.70	0.71–0.80	0.81–0.90	0.91–1.00	≥ 1.00
Rock number	13	41	50	32	27	10	7	9	5	3	3

Consequently, a set of classification standards was developed, taking into account the fragmentation degree, crack density, and the Engineering Rock Mass Classification Standard (GB/T 50218-2014)^13^:

1. Sparse crack: $$0 < \Gamma_{J} \le 0.4$$.
2. Slightly dense crack: $0.4 < \Gamma_{J} \le 0.6$.
3. Dense crack: $$0.6 < \Gamma_{J} \le 0.8$$.
4. Very dense crack: $0.8 \le \Gamma_{J} < 1.0$.
5. Crushed: $$\Gamma_{J} \ge 1.0$$.

**Figure 5:**
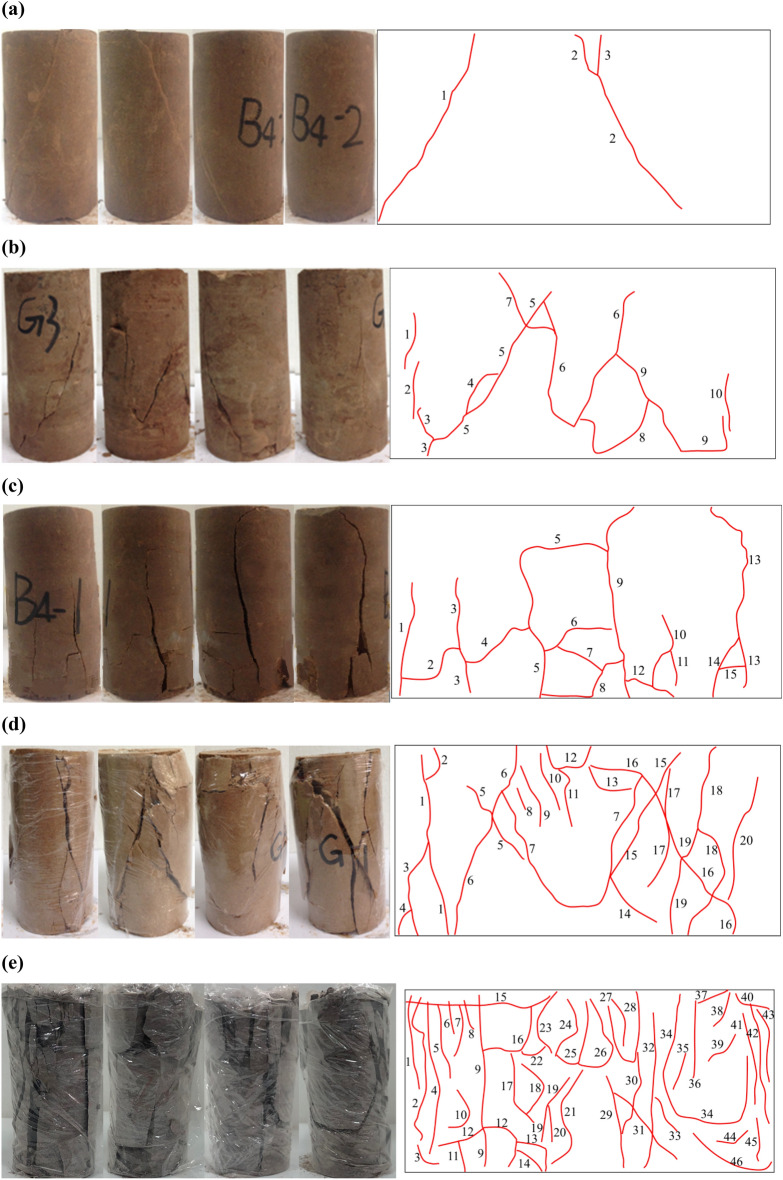
Crack density and fragmentation degree for typical rock samples: (**a**) 3 cracks, $\Gamma_{J} = 0.15$, category: sparse crack; (**b**) 10 cracks, $\Gamma_{J} = 0.50$, category: slightly dense crack; (**c**) 15 cracks, $\Gamma_{J} = 0.75$, category: dense crack; (**d**) 20 cracks, $\Gamma_{J} = 1.00$, category: very dense crack; (**e**) 46 cracks, $\Gamma_{J} = 2.30$, category: crushed.

Subsequently, the labeling process was carried out for the representative rock samples, as depicted in Fig. 5. Although the classification system based on crack density may have certain limitations, it was found to be consistent with the observed actual state of the rock samples, as shown in Fig. 5. Specifically, it was observed that the cracks in the rock samples did not propagate entirely from one tip to the other. Instead, a significant portion of the cracks consisted of short secondary cracks. Interestingly, it was observed that the degree of rock fragmentation was not necessarily similar for rock samples with the same number of short or long cracks, despite Eq. (1) yielding a similar crack density measurement. Therefore, in order to obtain a more realistic representation of crack density, it is imperative to consider the degree of crack propagation.

### Crack saturation

**Figure 6:**
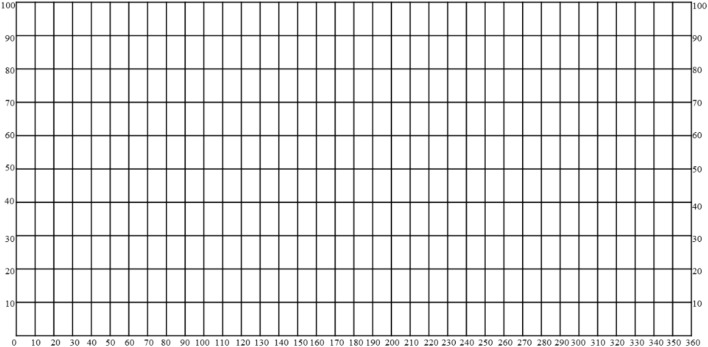
360 grids.

In practical applications, determining the exact degree of crack propagation poses a significant challenge. To simplify the analysis, a crack-saturation approach was employed, which involved considering the distribution of cracks within the rock samples. The crack propagation diagram utilized a grid-based system, consisting of 10 grids in the longitudinal direction and 36 grids in the lateral direction of the rock sample. Each grid had a spacing of 10 mm and 10°, resulting in a total of 360 grids in Fig. 6. The crack propagation was determined by counting the number of grids that contained cracks within the established grid-based system.

To construct the crack propagation diagram, the side of the rock sample was enveloped with 360 transparent grids, as illustrated in Fig. 6. Using a marking pen, the cracks observed in the rock sample were meticulously traced onto the transparent grids. Subsequently, the number of grids containing traced cracks was carefully counted to accurately quantify the extent of crack propagation.

To ensure consistency in the analysis, each grid was counted as having only one crack, even if multiple cracks appeared within the same grid. This approach simplified the counting process and provided a standardized measure of crack occurrence within each grid. Additionally, the crack saturation was defined as the ratio of the number of grids with cracks to the total of 360 grids, as demonstrated in Eq. (2).

2 $$\Gamma_{{\text{W}}} = \frac{{Q_{{\text{W}}} }}{360} \times 100{\text{\% }}{.}$$

In the given equation: $\Gamma_{{\text{W}}}$ represents the crack saturation and $Q_{{\text{W}}}$ denotes the number of grids with cracks.

**Figure 7:**
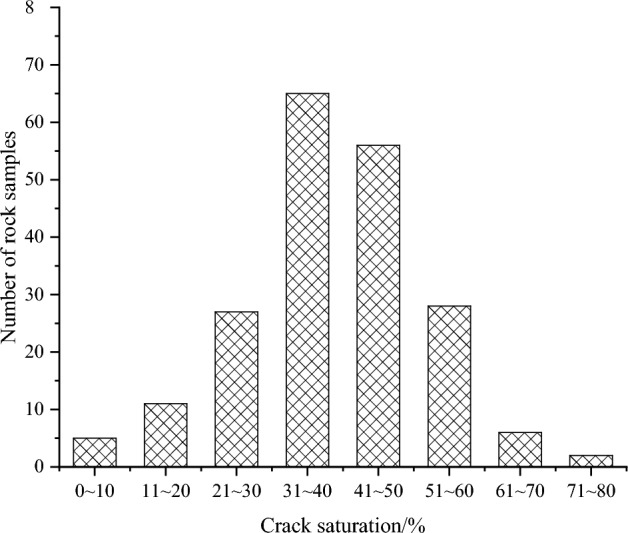
Histogram of crack saturation.

Accordingly, a histogram was constructed to illustrate the distribution of crack saturation across the 200 rock samples, as illustrated in Fig. 7. Based on the fragmentation degree, crack saturation, and the Engineering Rock Mass Classification Standard (GB/T 50218-2014)^13^, a comprehensive classification standard was developed, as shown below:

1. Integrity: $$0 < \Gamma_{W} \le 10\%$$.
2. Weakly fractured: $$10\% < \Gamma_{W} \le 30\%$$.
3. Fractured: $$30\% < \Gamma_{W} \le 50\%$$.
4. Strongly fractured: $$50\% < \Gamma_{W} \le 70\%$$.
5. Fully fractured: $$70\% < \Gamma_{W} \le 100\%$$.

**Figure 8:**
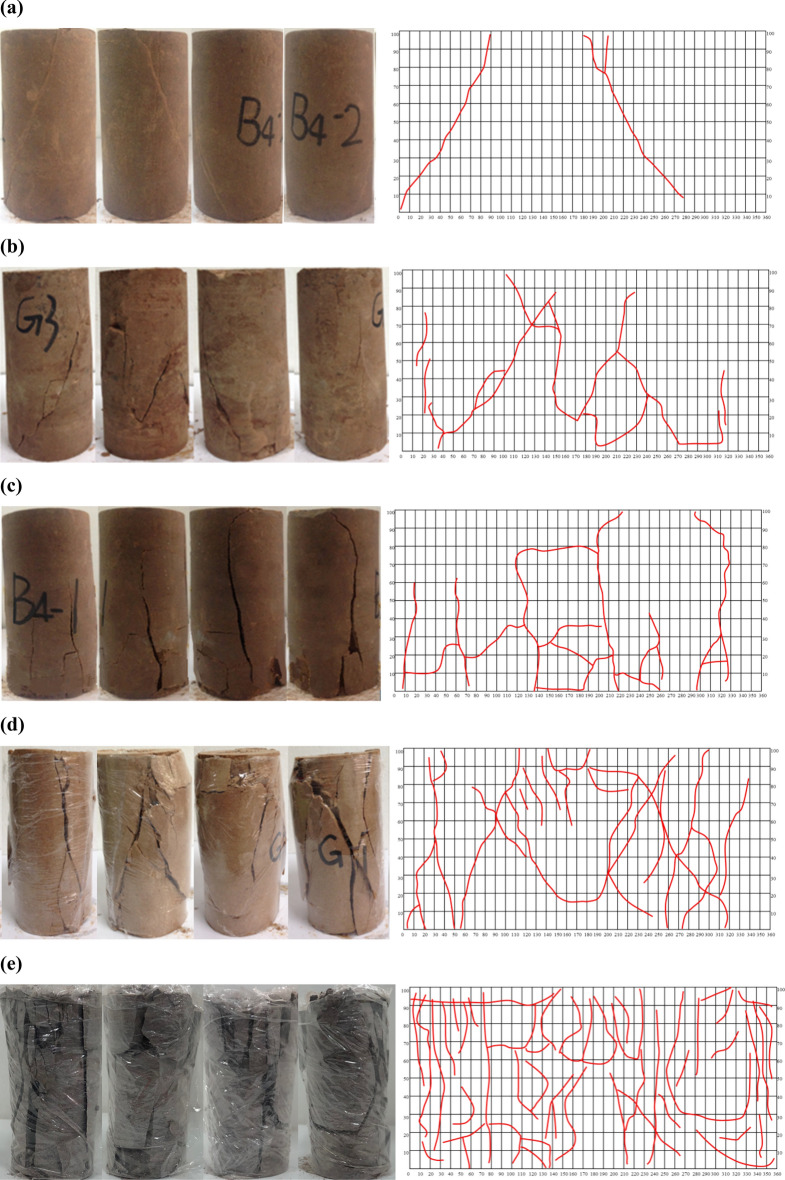
Crack saturation and fragmentation degree for typical rock samples: (**a**) 34grids,$\Gamma_{W} = 9.44\%$, category: integrity; (**b**) 82 grids, $\Gamma_{W} = 22.78\%$, category: weakly fractured; (**c**) 116 grids, $\Gamma_{W} = 32.22\%$, category: fractured; (**d**) 182 grids, $\Gamma_{W} = 50.56\%$, category: strongly fractured; (**e**) 256 grids, $\Gamma_{W} = 71.11\%$, category: fully fractured.

Based on the crack saturation analysis, Fig. 8 illustrates the degree of rock fragmentation for representative rock samples (as depicted in Fig. 5). While there is notable agreement between the classification system and the actual state depicted in Fig. 8, it is important to note that characterizing the fragmentation degree based solely on the saturation index may lead to inaccuracies. For example, it is important to recognize that rock samples exhibiting a few long cracks and several short cracks can have the same crack saturation value but differ in their fragmentation degrees.

## Degree of fragmentation based on crack density and saturation

The analysis conducted indicates that crack density and saturation can provide some insight into the fragmentation degree of rock samples. However, it is important to note that these results may not always be consistent. Rock samples with a few long cracks may exhibit a low crack density, but due to their presence in multiple grids, the crack saturation may be high. Conversely, rock samples with numerous short cracks may display a high crack density, but because of their limited occurrence within a few grids, the crack saturation may be low. This inconsistency can be observed in Table 3, where rows a and b demonstrate equal crack densities of 0.45, yet differing crack saturations of 22.78% and 9.44% due to the impact of crack length. Additionally, the two methods yield inconsistent results for the same rock sample type, as evident in rows b, c, and d of Table 3.

**Table 3:** Classification of rock fragmentation under different evaluation indexes.

Row number	Fractured rock samples	360 grid	Crack density $\Gamma_{{\text{J}}}$ and category	Crack saturation $\Gamma_{{\text{W}}}$ and category	Whether the classification levels are consistent
a			0.45 (slightly dense crack)	22.78% (weakly fractured)	Yes
b			0.45 (slightly dense crack)	9.44% (integrity)	No
c			0.25 (sparse crack)	12.78% (weakly fractured)	No
d			0.65 (dense crack)	27.50% (weakly fractured)	No

**Figure 9:**
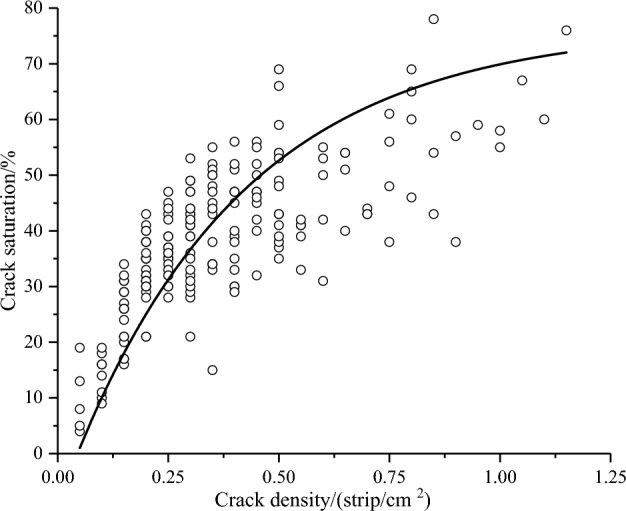
Relationship between crack density and crack saturation.

The relationship between crack density and crack saturation was found to be better represented by a power function, as depicted in Fig. 9, rather than a simple linear relationship. This suggests that the degree of fragmentation cannot be accurately characterized based solely on crack density or crack saturation alone.

Based on the analysis conducted, it is evident that crack density primarily represents the number of cracks present in a sample, rather than the extent of crack propagation. On the other hand, crack saturation provides a more accurate reflection of the actual crack propagation. These two evaluation indices, crack density and crack saturation, are complementary to each other in characterizing the degree of fragmentation in rock samples. Recognizing this complementarity, it becomes possible to develop a comprehensive evaluation method for the degree of fragmentation by combining these two indicators.

Taking into account the relationship between crack density and crack saturation, the degree of fragmentation was categorized utilizing Franklin’s rock classification system, as outlined in Table 4. To validate the universality of the proposed method, five rock samples were employed, and their results are presented in Table 5. The comprehensive classification method, which considers both crack density and crack saturation, effectively captured the actual fragmentation degree of the rock samples, as demonstrated in Table 5. This approach introduces a straightforward and convenient quantitative method for evaluating the degree of fragmentation.

**Table 4:**
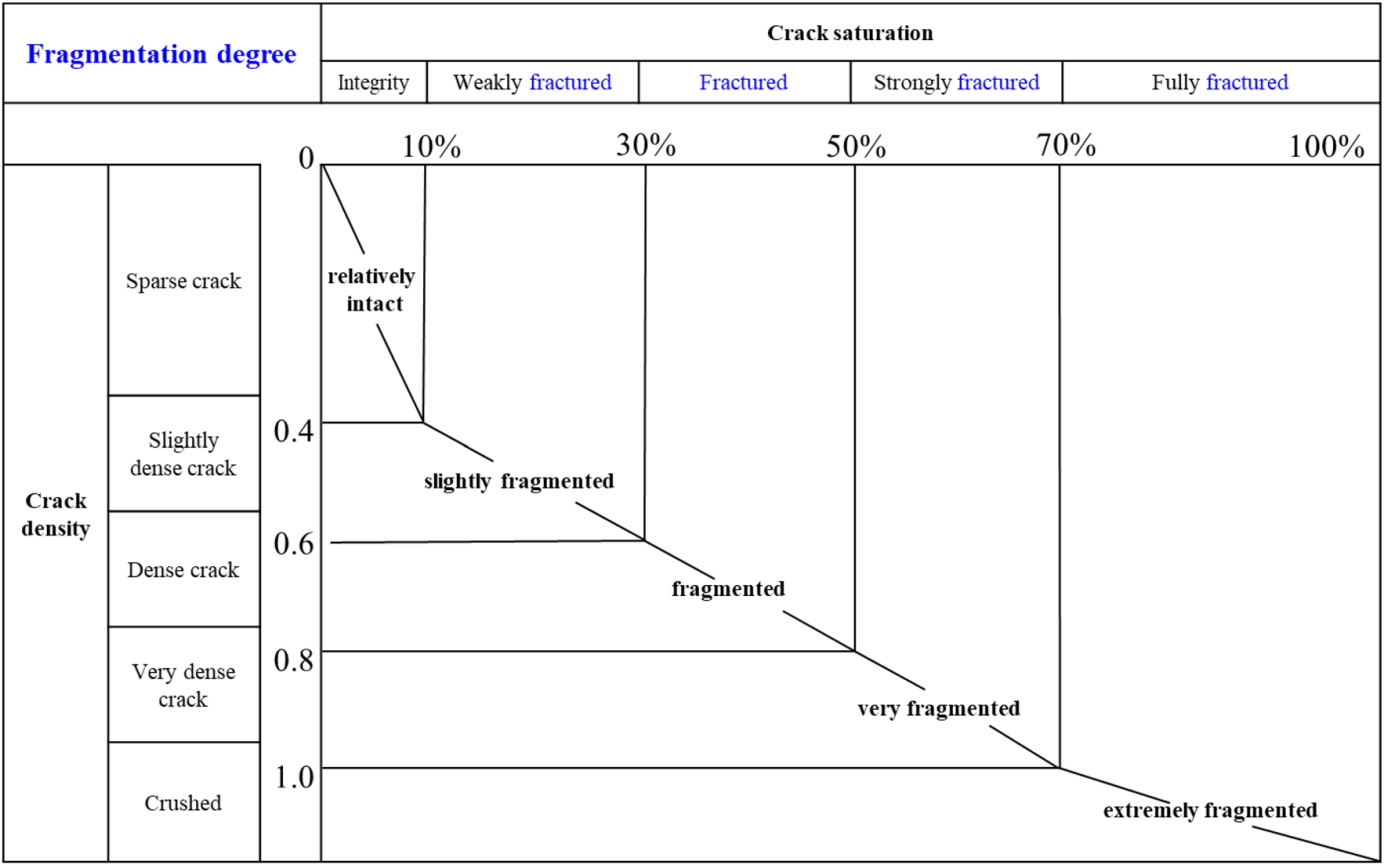
Classification table for rock fragmentation.

**Table 5:** Fragmentation degree evaluation for typical rock samples.

Fractured rock samples	360 grid	Crack density $\Gamma_{{\text{J}}}$	Crack saturation $\Gamma_{{\text{W}}}$	Fragmentation degree
		$0.2 \in \left( 0 \right.,\;\left. {0.40} \right]$	${8}.{33}\% \in \left( {0,\;\left. {10\% } \right]} \right.$	Relatively intact
		$0.{55} \in \left( {0.40,\;\left. {0.60} \right]} \right.$	${19}.{72}\% \in \left( {10\% ,\;\left. {30\% } \right]} \right.$	Slightly fragmented
		$0.{75} \in \left( {0.60,\;\left. {0.80} \right]} \right.$	${3}4.17\% \in \left( {30\% ,\;\left. {50\% } \right]} \right.$	Fragmented
		$0.95 \in \left( {0.80,\;\left. {1.00} \right]} \right.$	$50.56\% \in \left( {50\% ,\;\left. {70\% } \right]} \right.$	Very fragmented
		${2}.{6}0 \in \left( {1.00,\left. { + \infty } \right)} \right.$	${71}.{66}\% \in \left( {70\% ,} \right.\left. {100\% } \right]$	Extremely fragmented

## Discussion and conclusions

In the preceding sections, two indicators (crack density and crack saturation) were introduced to characterize the degree of rock fragmentation based on statistical analyses of 200 rock samples. Crack density serves as a measure of the number of cracks present within the rock sample. By considering crack density, the degree of rock fragmentation was classified into five categories: sparse crack, slightly dense crack, dense crack, very dense crack, and crushed. Furthermore, crack saturation emerged as a crucial factor in defining the propagation of cracks within the rock samples. Leveraging crack saturation, the rock fragmentation degree was further categorized into five classifications: integrity, weakly fractured, fractured, strongly fractured, and fully fractured. It is crucial to recognize that relying solely on crack density or crack saturation individually may result in inconsistencies when determining the expected fragmentation degree. These inconsistencies occur due to variations in the lengths of crack propagation within the rock samples.

A comprehensive rock classification method was developed using a combination of two indicators, crack density and saturation. This method categorizes the fragmentation degree into five distinct categories: relatively intact, slightly fragmented, fragmented, very fragmented, and extremely fragmented. The validation process demonstrated that the proposed classification method effectively captured the crack-propagation characteristics. It is important to note that while this study focused on standard cylindrical rock samples, the proposed evaluation method could be adapted for cubic samples as well, allowing for broader applicability in different experimental setups.

Furthermore, it is crucial to consider that this study was conducted using 200 rock samples. Future research should incorporate a larger dataset and consider additional statistical data and parameters to further refine and enhance the evaluation method. This would contribute to a more comprehensive and robust understanding of rock fragmentation characteristics.

## Data Availability

Some or all data, models, or codes generated or used during the study are available from the corresponding author upon request.
